# Natural medicines for radioprotection and radiosensitization: a bibliometric analysis of mechanisms and trends (2001–2025)

**DOI:** 10.3389/fonc.2026.1792854

**Published:** 2026-03-04

**Authors:** Yupeng Di, Jiazhao Song, Yingjie Wang, Lingling Meng, Jing Li

**Affiliations:** 1 Department of radiation protection medicine, School of Preventive Medicine, Fourth Military Medical University, Xi’an, China; 2 Ministry of Education Key Lab of Hazard Assessment and Control in Special Operational Environment, Xi’an, China; 3 Department of Radiation Oncology, Air Force Medical Center, People's Liberation Army, Beijing, China; 4 Experimental Tumorpathology, University Hospital Erlangen, Friedrich-Alexander-Universitat Erlangen-Nurnberg, Erlangen, Germany; 5 Department of Radiation Oncology, Senior Department of Oncology, The First Medical Center of People's Liberation Army General Hospital, Beijing, China

**Keywords:** bibliometric analysis, ionizing radiation, molecular mechanisms, natural medicines, radioprotection, radiosensitization

## Abstract

**Background:**

Ionizing radiation (IR) poses a global health threat, inducing molecular damage and chronic issues. Despite its significance, there has been limited bibliometric analysis to systematically evaluate the status, hotspots, and trends in the field of natural medicines (NMs) against IR.

**Purpose:**

To comprehensively understand the status, hotspots, and trends in the field of NMs against IR.

**Methods:**

Documents concerning NMs against IR were extracted from the Web of Science Core Collection (WoSCC) and PubMed databases. The literature analysis was conducted using VOSviewer 1.6.17 and CiteSpace 6.1.R6 software.

**Results:**

In total, 450 publications were encompassed. The most productive author was Baliga MS (19 documents) and Father Muller Medical College (11 documents, 688 citations); dominant countries were India (148 documents) and Peoples R China (99 documents); and a top journal was J Ethnopharmacol (615 citations). The first high-cited article was “Protection against Ionizing Radiation by Antioxidant Nutrients and Phytochemicals” by Weiss, JF (2003) with 459 citations. Oxidative stress, DNA damage, apoptosis, and radioprotection were identified as core research themes.

**Conclusion:**

Currently, the main hotspot is the elucidation of cellular and molecular mechanisms using novel technologies such as network pharmacology, molecular docking, and experimental validation. Future studies are needed to focus on the inherent molecular mechanisms and clinical applications. In addition, potential side effects of the bioactive compounds cannot be ignored.

## Introduction

Ionizing radiation (IR), owing to its widespread application in medical diagnostics and therapy, industrial processes, and its presence in environmental or accidental exposures, poses a significant threat to human health. It induces cellular and molecular damage, which can precipitate a range of acute and chronic health issues, including DNA damage, oxidative stress, inflammation, immune dysregulation, and an increased risk of cancer. Consequently, mitigating the adverse effects of IR on normal tissues while concurrently enhancing the efficacy of radiation therapy against cancer cells represents a critical challenge in modern medicine. In this context, radioprotection refers to the protection of healthy tissues from the harmful effects of radiation, whereas radiosensitization involves increasing the susceptibility of tumor cells to radiation therapy (1–4).

Natural medicines (NMs) possess a long history of therapeutic application and are highly regarded and widely utilized in many countries, including China, India, South Korea, and Japan. The diverse biological activities of NMs, such as their anti-tumor, anti-inflammatory, and anti-microbial properties, have been scientifically confirmed. Furthermore, their extracts and constituent compounds serve as valuable scaffolds for new drug design. In China, traditional Chinese herbs are integral to complementary and alternative therapies due to their promising physiological effects and low toxicity profiles. The therapeutic potential of traditional herbal medicine has been recognized by the World Health Organization (WHO), which initiated a program from 2014 to 2023 to promote and investigate their safety, efficacy, and quality standards, encouraging broader international adoption. It is believed that the chemical components of medicinal plants, with their wide-ranging applications, can lead to the development of innovative products with fewer side effects than existing medications (5–8).

A substantial body of research has demonstrated that NMs such as *Panax ginseng* (Ginseng), *Hippophae rhamnoides* (Sea buckthorn), Triphala (a polyherbal formulation), and *Emblica officinalis* (Amla), along with various plant extracts and phytochemicals, exhibit distinct protective effects against IR-induced cellular damage. These effects include reducing oxidative stress, modulating immune responses, and enhancing the radiosensitivity of cancer cells. Similarly, natural bioactive molecules—including ferulic acid, mangiferin, naringenin, resveratrol, isofraxidin, eugenol, honokiol, arbutin, paeoniflorin, chlorogenic acid, and quinic acid—have been identified for their potential radioprotective or radiosensitizing properties. Over recent decades, extensive research has explored the mechanisms by which NMs prevent and manage IR-induced damage, endorsing the therapeutic benefits of natural compounds and single agents. For instance, studies have reported that traditional Chinese medicine (TCM) compounds or extracts can mitigate radiation-induced injuries to the bone marrow, intestines, testes, and liver (9–12).

In contrast to traditional literature reviews, bibliometric analysis provides a quantitative assessment of a research field by evaluating its literature characteristics through visualizing processing tools like CiteSpace and VOSviewer. With the rapid accumulation of data, identifying high-value research directions from the vast volume of literature has become increasingly difficult. This approach enables the identification of predominant institutions and countries, leading authors and journals, top-cited references, and emerging research trends or hotspots. Given the significant role of NMs in mitigating the effects of IR and the absence of a recent comprehensive bibliometric study on this topic, this research aims to delineate publication trends and identify significant hotspots by analyzing literature published from 2001 to 2025.

## Materials and methods

### Literature sources and retrieval strategy

**Figure 1:**
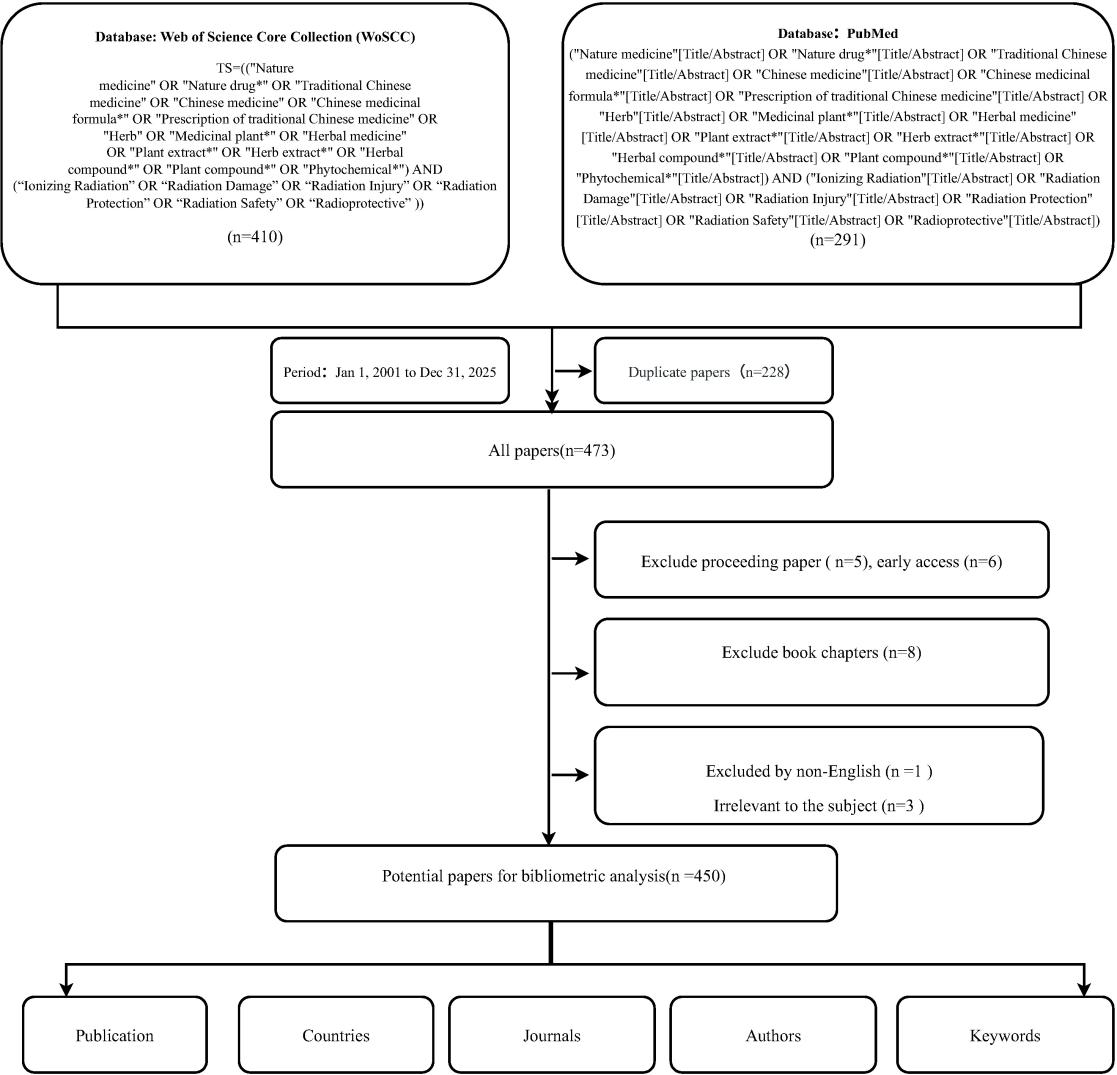
Flowchart of the literature selection process.

For this bibliometric analysis, the Web of Science Core Collection (WoSCC) and PubMed databases were utilized as the primary data sources. The retrieval strategy was formulated based on specific topic searches. For WoSCC, the field tag “TS” (Topic) was used, and for PubMed, the tag “[Title/Abstract]” was applied to ensure precision. The search queries combined keywords related to natural medicines (e.g., “Nature medicine,” “Traditional Chinese medicine,” “Phytochemical”) and ionizing radiation (e.g., “Ionizing Radiation,” “Radiation Protection,” “Radioprotective”). The specific search strings are detailed in **Figure 1**.

The search period was from January 1, 2001, to December 31, 2025. The initial search yielded a total of 701 records, comprising 410 from WoSCC and 291 from PubMed. Subsequently, 228 duplicate records were identified and removed, resulting in a dataset of 473 unique publications. As depicted in **Figure 1**, a detailed exclusion process was then applied to these 473 papers. Book chapters (n=8), proceeding papers (n=5), and early access publications (n=6) were excluded from the dataset. Furthermore, publications were screened to exclude non-English papers (n=1) and articles irrelevant to the subject (n=3). Finally, a total of 450 publications were identified as eligible for bibliometric analysis.

### Data collection and analysis methods

The full records and cited references were exported from the WoSCC database in plain text format. VOSviewer software (version 1.6.17) was used to perform co-occurrence analysis of all keywords and to map the co-authorship networks of organizations, authors, and countries. For co-cited authors, references, and journals, the minimum appearance thresholds were set to 30, 13, and 120, respectively. For co-authorship analysis, the minimum appearance frequencies for authors, organizations, and countries were set at 5, 5, and 3, respectively.

CiteSpace software (version 6.1.R6) was employed to conduct co-occurrence analysis and burst detection of keywords. The time range for this analysis was set from January 2001 to December 2025, with each time slice representing one year. The cosine algorithm was used to calculate relationship strength links. The Pathfinder algorithm was selected for network pruning, with the “Pruning networks” method as an auxiliary strategy; all other settings were kept at their defaults. Subsequently, cluster analysis was performed on the keyword co-occurrence network, and the log-likelihood rate (LLR) algorithm was used to extract cluster tags (13–15).

## Results

### Trends of annual publications

**Figure 2:**
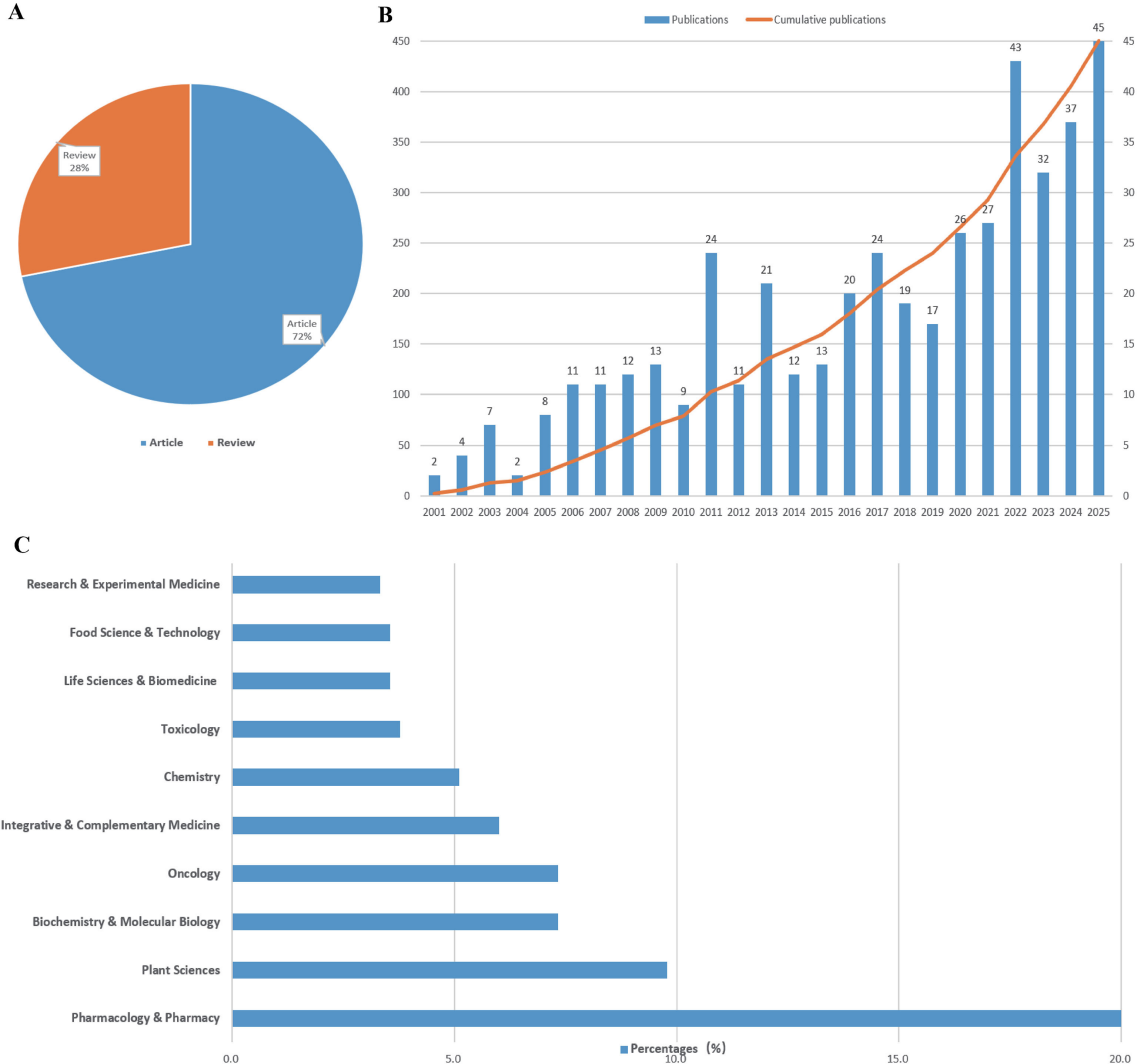
Trends in publications on natural medicines against ionizing radiation from 2001 to 2025. **(A)** Distribution of literature types, **(B)** Annual publication quantity and cumulative growth, and **(C)** Distribution of subject categories.

A total of 450 research articles and reviews in English were included in the final analysis. As presented in **Figures 2A**, the majority of publications were articles (72%), while reviews constituted the remaining 28%. The annual publication output on NMs against IR demonstrates a marked increasing trend, with a corresponding cumulative growth over the study period (**Figure 2B**). This growth was particularly pronounced from 2018 onwards, with 2022, 2023, and 2024 showing high publication counts of 43, 37, and 45 publications, respectively. This surge indicates a growing recognition of and research interest in this topic. Analysis of subject categories (**Figure 2C**) reveals the interdisciplinary nature of this field, with the top five categories being Pharmacology & Pharmacy (20.0%), Plant Sciences (9.8%), Biochemistry & Molecular Biology (7.3%), Oncology (7.3%), and Integrative & Complementary Medicine (6.0%).

### Cooperation networks between countries, institutions, and authors

**Figure 3:**
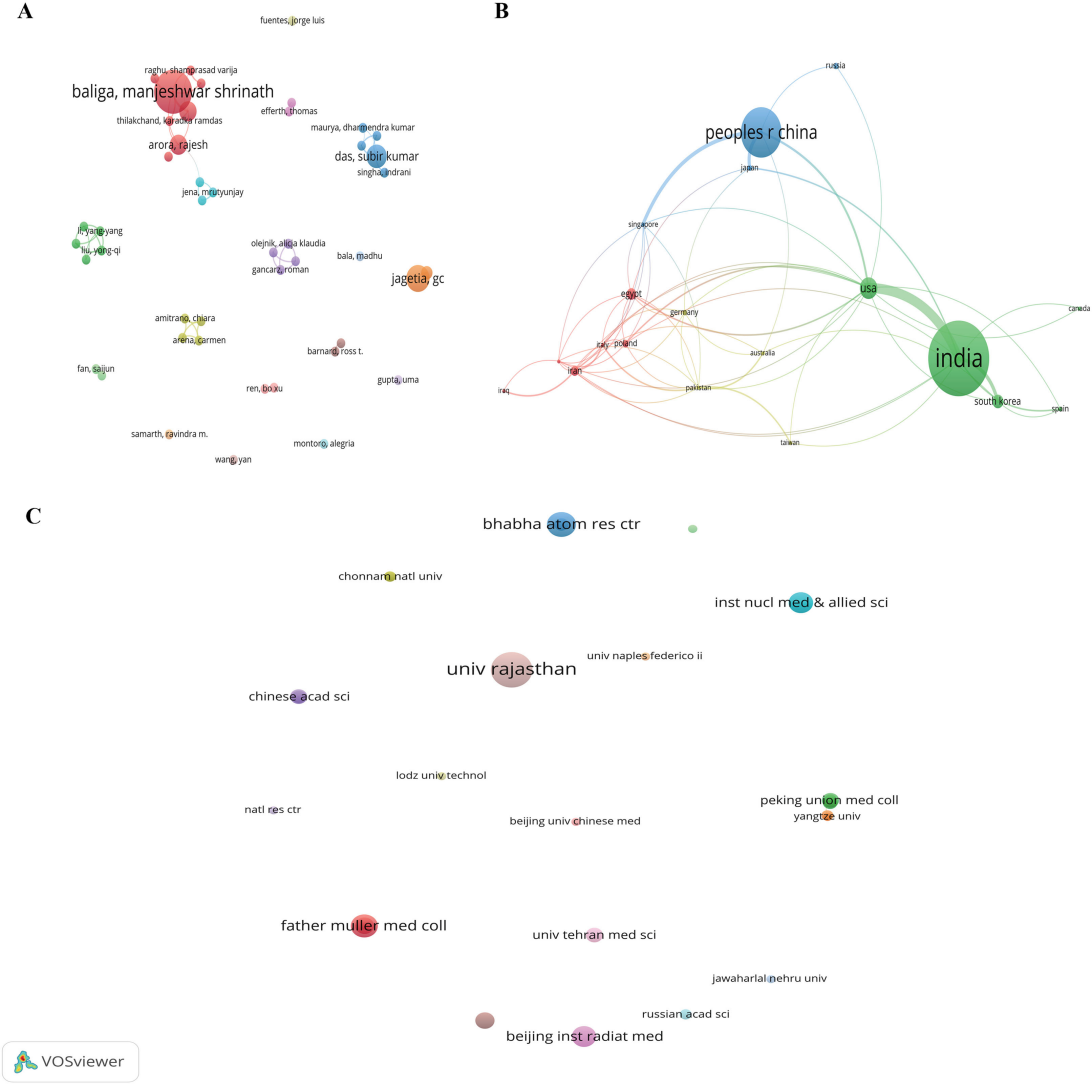
Co-authorship networks in the field of natural medicines against ionizing radiation. **(A)** Co-authorship map of authors, **(B)** Co-authorship map of countries, and **(C)** Co-authorship map of organizations.

The field of NMs against IR is characterized by extensive collaboration among numerous authors, institutions, and countries. The author co-authorship network (**Figure 3A**) highlights key researchers such as Jagetia, GC, Baliga, MS, Samarth, RM, and Weiss, JF, who appear prominently within collaborative clusters, indicating their significant roles in driving research.

Analysis of national contributions (**Figure 3B**) reveals that India is the most prolific country, with 148 publications and 3813 citations, followed by the People’s Republic of China (99 publications, 1670 citations) and the USA (43 publications, 1797 citations). These nations have established themselves as the most active in the field, with India in particular displaying a robust network of both internal and external collaborations.

The co-authorship map of organizations (**Figure 3C**) shows that the most productive institutions are predominantly from India and China. For instance, the University of Rajasthan (India) led with 17 documents and 349 citations, followed by the Bhabha Atomic Research Centre (India) with 12 documents and 227 citations. Notably, Father Muller Medical College (India) published 11 documents that garnered a remarkable 688 citations. Other significant contributors include the Institute of Nuclear Medicine & Allied Sciences (India), Kasturba Medical College & Hospital (India), and the Beijing Institute of Radiation Medicine (China), each with 10 documents. The high average citation rates for institutions like Father Muller Medical College and Kasturba Medical College & Hospital underscore the impactful nature of their research (**Table 1**).

**Table 1:** The top 10 cooperative institutions regarding natural medicines against IR.

Rank	Institutions	Country	Documents	Citations	Average citations
1	University of Rajasthan	India	17	349	20.53
2	Bhabha Atomic Research Centre	India	12	227	18.92
3	Father Muller Medical College	India	11	688	62.55
4	Institute of Nuclear Medicine & Allied Sciences	India	10	162	16.20
5	Kasturba Medical College & Hospital	India	10	422	42.20
6	Beijing Institute of Radiation Medicine	China	10	111	11.10
7	Mangalore Institute of Oncology	India	8	208	26.00
8	Atomic Energy Authority	India	8	95	11.88
9	Peking Union Medical College	China	7	183	26.14
10	Chinese Academy of Sciences	China	7	86	12.29

### Citation/co-citation analysis of authors, journals, and references

**Figure 4:**
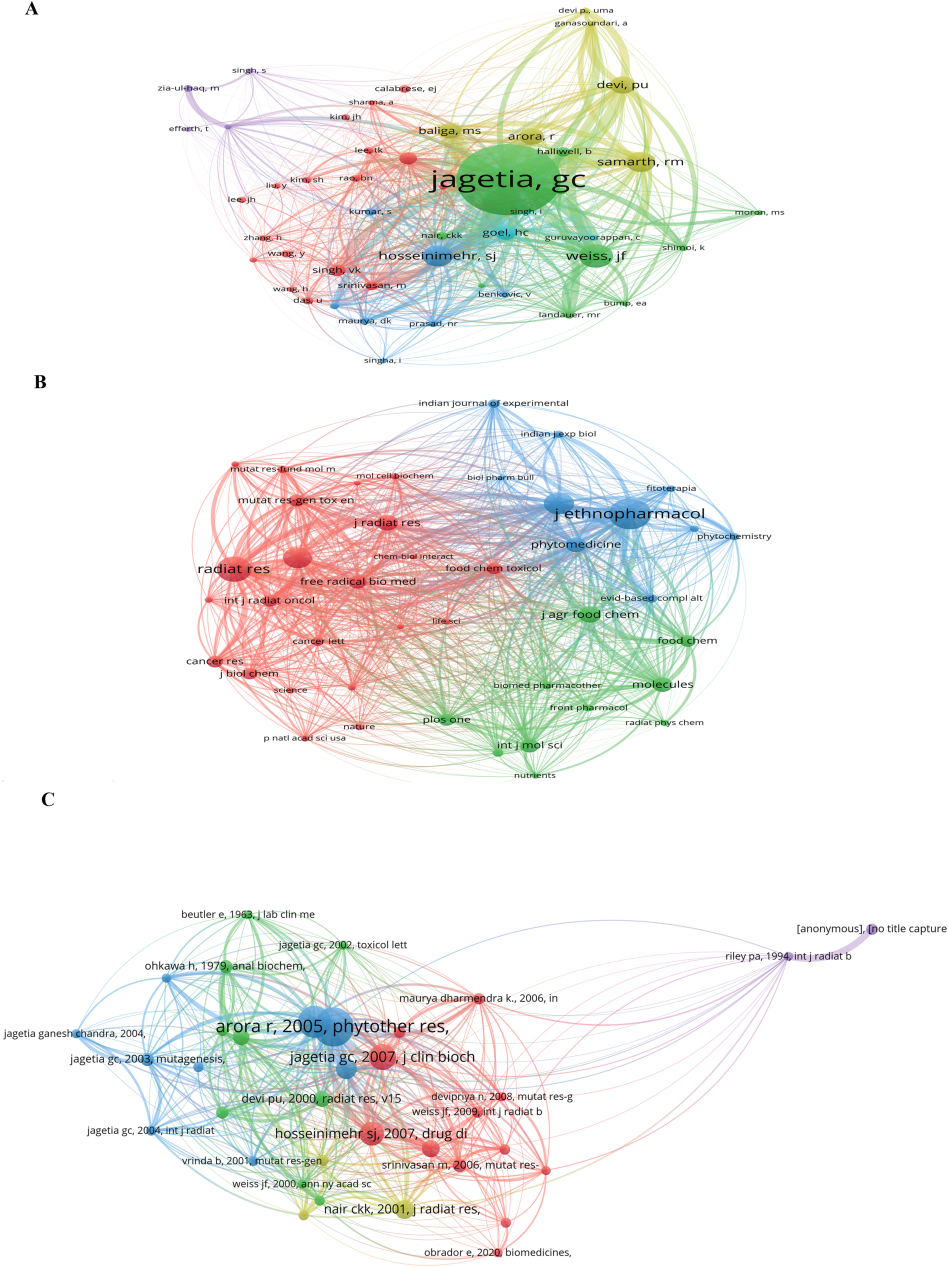
Co-citation analysis of publications on natural medicines against ionizing radiation. **(A)** Co-citation network of authors, **(B)** Co-citation network of journals, and **(C)** Co-citation network of references.

Citation analysis serves as a reliable indicator of the quality and impact of scholarly work. The co-citation network for authors, visualized in **Figures 4A**, identifies Jagetia, GC (294 citations), Weiss, JF (97 citations), Hosseinimehr, SJ (87 citations), and Samarth, RM (83 citations) as central and highly influential figures whose work forms the intellectual foundation of the field.

Over the past two decades, a multitude of scholarly journals have published articles related to NMs against IR. Based on total citations, the leading journals include *J Ethnopharmacol* (615 citations), *Radiat Res* (504 citations), *Phytother Res* (439 citations), and *Int J Radiat Biol* (421 citations), as depicted in **Figure 4B**. These journals are distinguished by their high citation counts, reflecting their significant influence. The majority of these top-tier journals are classified in the Q1 or Q2 JCR divisions, testifying to their strong academic standing. Although India is a leading contributing country, the premier journals in this field are primarily based in Europe and the United States (**Table 2**).

**Table 2:** The top 10 most active journals in research of natural medicines against IR (sorted by total citation) from 2001 to 2025.

Rank	Journal	Documents	Total citations	Average citations per paper	IF (2025)	JCR division	Country
1	Journal of Ethnopharmacology	12	575	47.92	5.4	Q1	Netherlands
2	Phytomedicine	11	621	56.45	8.3	Q1	Germany
3	International Journal of Radiation Biology	10	130	13.00	2.4	Q1	UK
4	Biomedicine & Pharmacotherapy	9	140	15.56	7.5	Q1	France
5	Phytotherapy Research	9	337	37.44	6.3	Q1	UK
6	Evidence-Based Complementary and Alternative Medicine	8	130	16.25	2.6	Q2	UK
7	International Journal of Molecular Sciences	8	120	15.00	4.9	Q1	Unite States
8	Indian Journal of Experimental Biology	7	269	38.43	0.5	Q4	India
9	Molecules	5	214	42.80	4.6	Q2	Switzerland
10	Frontiers in Pharmacology	5	160	32.00	4.8	Q2	Switzerland

**Figure 4C** illustrates the network of co-cited references, highlighting foundational publications. The most highly cited works include comprehensive reviews and seminal experimental studies that have profoundly shaped the understanding of radioprotective mechanisms. For instance, the articles “Protection against Ionizing Radiation by Antioxidant Nutrients and Phytochemicals” by Weiss, JF (2003) and the work by Arora, R (2005) in *Phytotherapy Research* are among the most frequently co-cited, demonstrating their enduring influence (1, 16). The top 10 globally cited references are listed in **Table 3**, with the work by Weiss (2003) ranking first.

**Table 3:** Top 10 highest cited references in natural medicines against IR from 2001 to 2025.

Rank	Title	Journal	Total citations	Publication year	First author
1	Protection against Ionizing Radiation by Antioxidant Nutrients and Phytochemicals	Toxicology	459	2003	James F. Weiss
2	Antioxidant Health Effects of Aged Garlic Extract	The Journal of Nutrition	353	2001	Carmia Borek
3	Medicinal and therapeutic potential of Sea buckthorn (Hippophae rhamnoides L.)	Journal of Ethnopharmacology	347	2011	G. Suryakumar
4	Effects of Radiation Processing on Phytochemicals and Antioxidants in Plant Produce	Trends in Food Science & Technology	204	2009	Mohammed Alothman
5	Evaluation of antioxidant and radical-scavenging activities of certain radioprotective plant extracts	Food Chemistry	188	2008	RM Samarth
6	Biological Activities of Crude Extracts and Chemical Constituents of Bael, Aegle Marmelos (I.) Corr.	Indian Journal of Experimental Biology	181	2009	Prabir Maity
7	The evaluation of the radioprotective effect of Triphala (an ayurvedic rejuvenating drug) in the mice exposed to gamma-radiation	Phytomedicine	166	2002	Ganesh Chandra Jagetia
8	Radioprotective potential of ginseng	Mutagenesis	151	2005	Tae Kyoung Lee
9	Ethnomedicinal, phytochemical and pharmacological updates on Peppermint (Mentha × piperita L.)-A review	Phytotherapy Research	145	2020	Ganapathy Mahendran
10	Artesunate induces oxidative DNA damage, sustained DNA double-strand breaks, and the ATM/ATR damage response in cancer cells	Molecular Cancer Therapeutics	144	2011	Nadine Berdelle

**Figure 5:**
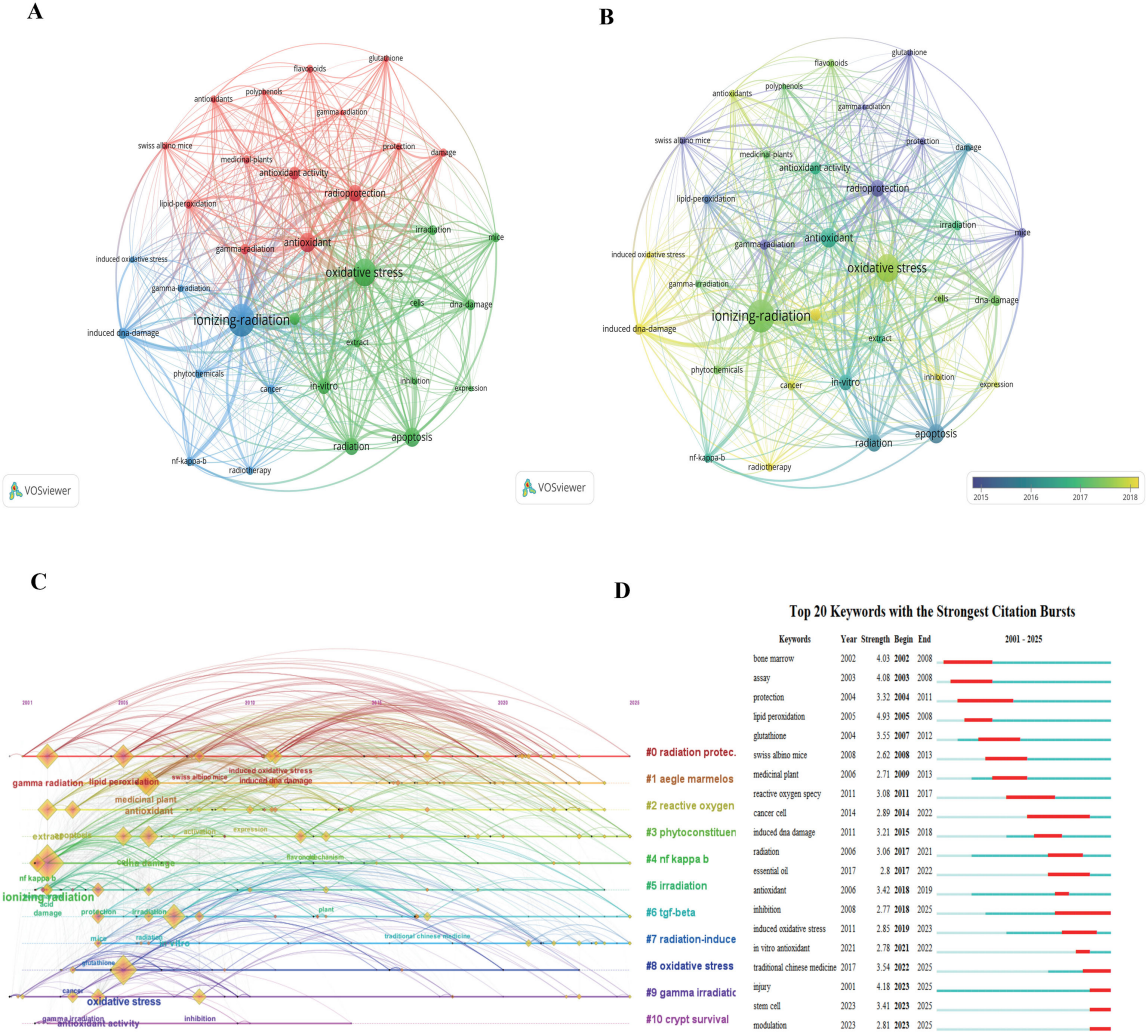
Keyword analysis in the field of natural medicines against ionizing radiation. **(A)** Keyword co-occurrence network, **(B)** Keyword overlay visualization, **(C)** Keyword timeline view, and **(D)** Top 20 keywords with the strongest citation bursts.

Furthermore, an analysis of reference citation bursts using CiteSpace (**Figure 5D**) indicates emerging or sustained research interest in specific topics. Keywords such as “radiation protection,” “oxidative stress,” and “DNA damage” show strong and prolonged citation bursts, underscoring their foundational importance.

### Keywords analysis

#### Co-occurrence network analysis of keywords

Keywords provide a concise summary of a research field’s focus. The co-occurrence network of keywords was analyzed using VOSviewer, with the results visualized in **Figures 5A, B**. Frequently used keywords include oxidative stress, inflammation, DNA damage, apoptosis, and the names of various plants and their active compounds.

As depicted in **Figures 5A**, the keywords form three main clusters. Cluster 1 (red) primarily focuses on antioxidant defense and radioprotection, with terms such as “antioxidant,” “flavonoids,” “gamma radiation,” “glutathione,” “lipid peroxidation,” and “radioprotection.” Cluster 2 (green) centers on cellular responses to radiation and damage mechanisms, including “apoptosis,” “cells,” “DNA damage,” “ionizing radiation,” and “oxidative stress.” Cluster 3 (blue) relates to cancer and specific molecular targets, featuring keywords like “cancer,” “induced DNA damage,” “NF-kappa-b,” “phytochemicals,” and “radiotherapy.” The overlay visualization in **Figure 5B** illustrates the temporal evolution of these keywords. Foundational terms like “ionizing radiation” and “radiation protection” (in blue/purple) were prominent in earlier years, while more recent attention (in yellow/green) has shifted towards concepts such as “oxidative stress,” “DNA damage,” “apoptosis,” “radiosensitization,” and specific compounds like “polyphenols” and “flavonoids.”

The timeline view (**Figure 5C**) further illustrates that keywords related to radiation protection and oxidative stress have been consistent hot topics since the early 2000s. Clusters focusing on “DNA damage,” “apoptosis,” and “anti-inflammatory” effects also show sustained research interest. Meanwhile, emerging concepts such as “radiosensitization” and “nanoparticles” signify newer research directions. The keyword burst analysis (**Figure 5D**) confirms the importance of terms like “radiation protection,” “oxidative stress,” “DNA damage,” “apoptosis,” and “radiosensitization,” all of which exhibit significant and sustained bursts. More recently, “nanoparticles” and “molecular docking” also show strong bursts, indicating their growing relevance as modern tools for elucidating the mechanisms of NMs against IR.

## Discussion

This study presents a bibliometric analysis of the applications of NMs against IR from 2001 to 2025, using the WoS core collection and visualized with VOSviewer and CiteSpace software. The intuitive display of annual publication numbers, country and institutional collaborations, influential authors and journals, and keyword trends offers a comprehensive overview of global research hotspots, providing a valuable reference for researchers in this field.

### Research overview and characteristics of publications

The analysis confirms a growing interest in NMs as countermeasures to IR. While research output was relatively modest from 2001 to 2011, the rate of publication accelerated significantly after 2012, with a notable surge from 2018 onwards (**Figure 2B**). This growth is likely attributable to advancements in molecular biology techniques, the advent of network pharmacology and bioinformatics methods, and an increasing global demand for effective and less toxic radioprotective and radiosensitizing agents (7, 8).

The co-authorship analysis of countries (**Figure 3B**) clearly establishes India and China as the dominant forces in this research domain, with India leading significantly in publication volume and citation impact. This leadership is likely fueled by their rich heritage in traditional medicine and substantial governmental support. As revealed by **Figures 3C**, the most productive institutions are predominantly from these two nations (**Table 1**), including the University of Rajasthan, Bhabha Atomic Research Centre, and Father Muller Medical College in India, and the Beijing Institute of Radiation Medicine in China. The high academic impact of these institutions underscores their excellent research capabilities. To further advance the field, enhanced collaboration between scholars and institutions worldwide is encouraged.

The 450 articles analyzed were published in numerous scholarly journals. As indicated in **Figures 4B**, journals such as *J Ethnopharmacol*, *Radiat Res*, *Phytother Res*, and *Int J Radiat Biol* are among the most active and highly cited venues (**Table 2**). The prominence of these high-impact journals, many of which are in the Q1 and Q2 JCR divisions, signifies the high academic standing and quality of research in this field.

### Research trends and hot spots on NMs against IR

Analysis of cited references and keywords (**Figure 4**, **Figure 5**) reveals a clear developmental trend. The field has evolved from initial studies on the general efficacy of traditional medicines to rigorous investigations into the cellular and molecular mechanisms of radiation damage and protection, alongside the identification of specific active compounds using advanced technologies. Keywords such as network pharmacology and molecular docking show consistent and recent citation bursts (**Figure 5D**), reflecting a strong interest in systematic approaches to unravel complex pharmacological interactions. Other prominent hotspots include oxidative stress, inflammation, DNA damage, apoptosis, and radiosensitization, indicating a comprehensive focus on understanding and manipulating the biological responses to IR. The emergence of terms like nanoparticles suggests a new frontier in drug delivery and therapeutic efficacy (2, 4, 17, 18).

### Common NMs used in IR

A diverse array of natural medicines has been extensively studied for their efficacy against IR. Among these, herbal medicines and their extracts are prominent. Ginseng is widely investigated for its radioprotective properties, including preventing radiation-induced DNA damage and reducing myelosuppression, liver injury, and apoptosis, with its active ginsenosides mediating these effects (10, 19, 20). Similarly, Triphala, an Ayurvedic polyherbal drug, has been shown to reduce radiation-induced mortality and protect against gastrointestinal and hematopoietic damage, primarily via antioxidant mechanisms (21). Sea Buckthorn is explored for its broad medicinal potential, including cytoprotective, anti-stress, and radioprotective effects (9). Emblica officinalis, or Indian gooseberry, shows potent radioprotective and chemopreventive effects through free radical scavenging and antioxidant activities (11, 22). Other notable herbs include *Aegle marmelos* (Bael), recognized for its antioxidant and radioprotective properties (23–25); *Ophiocordyceps sinensis* (formerly *Cordyceps sinensis*), which protects against bone marrow and intestinal injuries by reducing ROS (26); and *Zingiber officinale* (Ginger), whose rhizomes and phytochemicals possess radioprotective effects (12). *Tinospora cordifolia* (Guduchi) ameliorates radiation-induced testicular injury, while *Mentha piperita* (Peppermint) safeguards various radiosensitive organs through multiple mechanisms, including antioxidant activity and enhanced DNA repair (27–29). *Angelica sinensis* (Radix Angelica Sinensis) is used in TCM for various medicinal effects, including hematopoietic, antioxidant, immunoregulatory, and radioprotective activities (30). Traditional Chinese Medicine formulas like Bu-Zhong-Yi-Qi-Tang and Wuzi Yanzong pill have demonstrated protective effects on hematopoietic organs and testicular tissue, respectively (31, 32). Xuebijing injection also mitigates hematopoietic cell injury by decreasing ROS levels (33). *Moringa oleifera* (Drumstick tree) leaf extract ameliorates ionizing radiation-induced lipid peroxidation in mouse liver, with phytochemicals like ascorbic acid, phenolics contributing to its antioxidant effects (34). *Grewia asiatica* (Phalsa) fruit exhibits strong free radical scavenging activity and radioprotective efficacy, protecting against lipid peroxidation and DNA damage (35).

Furthermore, specific bioactive compounds isolated from these plants are at the forefront of research. Ferulic acid protects hepatocytes from radiation-induced damage, while chlorogenic acid and quinic acid shield human lymphocytes from DNA damage (36, 37). Mangiferin protects against radiation-induced sickness and mortality, and naringenin offers protection against DNA, chromosomal, and membrane damage by inhibiting the NF-κB pathway (38, 39). Resveratrol has demonstrated radioprotective activity against chromosomal damage (40). Isofraxidin, a coumarin compound, protects leukemia cells from apoptosis via the ROS/mitochondria pathway (41). Other compounds, such as eugenol, honokiol, and artesunate, show promise as radiosensitizers by inducing apoptosis and targeting key signaling pathways in cancer cells (3, 42, 43). Arbutin, an intracellular hydroxyl radical scavenger, protects cells from radiation-induced apoptosis (44). Paeoniflorin protects thymocytes by scavenging ROS and modulating MAP kinase pathways (45). Celastrol potentiates radiotherapy by impairing DNA damage processing, and berberine enhances radiosensitivity by targeting HIF-1α (46, 47). Finally, compounds like orientin and polysaccharide-polyphenolic conjugates are recognized for their broad radioprotective and antioxidant activities (6, 48).

### New technologies or approach applied in the field of NMs against IR

Network pharmacology, a discipline grounded in systems biology, analyzes the complex interactions between drug components and multiple targets. This approach, which elucidates how drugs act on disease-related signaling modules, has become a promising tool for revealing the pharmacological mechanisms of traditional medicine formulas. It has been increasingly used to identify potential therapeutic targets of NMs and to understand their molecular mechanisms in combating IR-induced damage and enhancing radiosensitivity (30, 48).

To further clarify these complex mechanisms, researchers are increasingly combining network pharmacology with other approaches, such as molecular docking and experimental verification. Molecular docking is widely used to predict the binding conformation and free energy of small molecules to their protein targets. These integrated strategies have enabled more precise studies of the therapeutic mechanisms of NMs against radiation injury and cancer radioresistance by identifying specific molecular targets and pathways. The integration of comprehensive metabolomics with network pharmacology is also being used to reveal the metabolic impact and mechanisms of NMs in treating radiation-related conditions (49).

>Additionally, the simultaneous application of network pharmacology, molecular docking, and experimental verification has become a powerful methodology for revealing the mechanisms of traditional formulas in treating acute injuries and enhancing cancer therapy. This multi-omics approach facilitates the identification of active components, protein targets, and relevant signaling pathways, such as the PI3K/AKT pathway, within the context of cellular responses to radiation (20).

### A summary of the inherent molecular mechanisms

**Figure 6:**
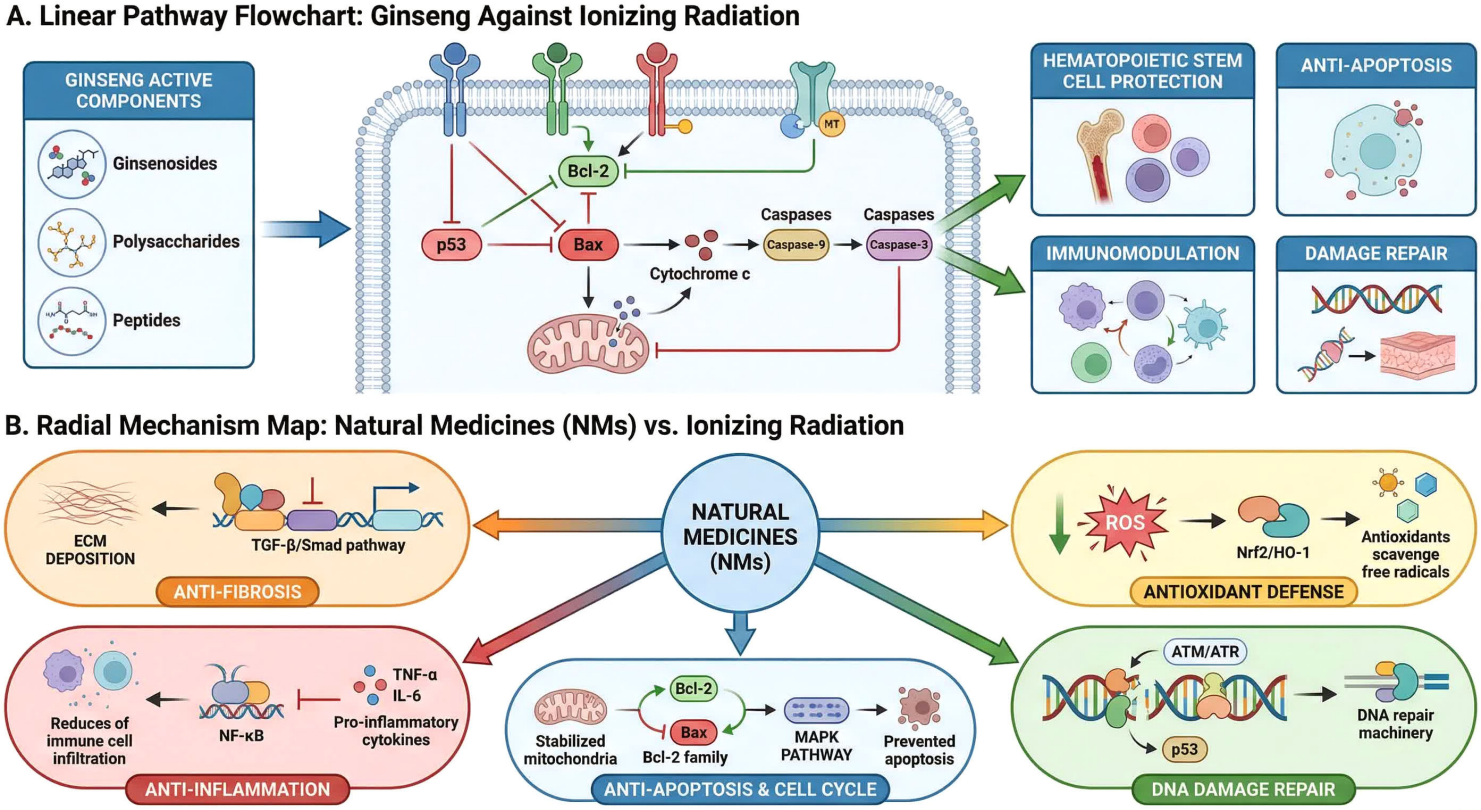
Proposed molecular mechanisms of natural medicines against ionizing radiation. **(A)** Illustration of multi-targeted mechanisms, exemplified by ginseng. **(B)** Summary of various interconnected molecular mechanisms involved in the protective functions of natural medicines.

As summarized in **Figures 6B**, the protective functions of NMs against IR are mediated by several interconnected molecular mechanisms. Primarily, NMs provide robust antioxidant defense by directly scavenging reactive oxygen species (ROS), neutralizing free radicals, reducing lipid peroxidation, and activating the NRF2 pathway. NRF2 activation, in turn, upregulates key antioxidant enzymes such as HO-1, GPx, and SOD1, thereby enhancing the cell’s endogenous antioxidant capacity. In addition to their antioxidant activity, NMs exhibit significant anti-inflammatory properties. IR often triggers inflammatory responses through the production of pro-inflammatory cytokines and the activation of transcription factors like NF-κB. NMs can effectively block NF-κB activation and suppress the release of these inflammatory mediators. Furthermore, NMs play a crucial role in DNA damage repair and cell-cycle regulation, contributing to the maintenance of genomic stability. In terms of anti-fibrotic effects, which can be a long-term consequence of radiation, NMs help mitigate this process by inhibiting signaling pathways like TGF-β/Smad, modulating MAPK/JNK/ERK pathways, reducing the deposition of connective tissue growth factor (CTGF) and extracellular matrix (ECM), and restoring the balance of tissue remodeling enzymes. As illustrated by the example of ginseng in **Figures 6A**, NMs often employ a multi-targeted approach, which includes modulating apoptosis via the p53/Bax/Bcl-2 axis, enhancing immune responses, protecting hematopoietic stem cells, and ultimately reducing inflammation and tissue damage (50–53).

### Limitations

This bibliometric study has systematically analyzed the basic situation, research hotspots, and trends regarding the effects of NMs against IR from a visualization perspective. The results provide valuable insights for researchers already in or interested in this field. However, it is crucial to acknowledge certain inherent limitations. Firstly, distinct biomedical databases such as EMBASE, COCHRANE, CINAHL, and PROQUEST were not included in this analysis. This study primarily relied on the WoSCC database because it offers the most comprehensive and standardized citation data required for the algorithms of VOSviewer and CiteSpace. While the excluded databases contain valuable literature, merging data from disparate sources often introduces significant inconsistencies in citation counting and metadata formats, which can compromise the accuracy of the bibliometric mapping. Secondly, the analytical algorithms employed by VOSviewer and CiteSpace, while powerful, inherently involve certain processing parameters and thresholds (e.g., minimum co-occurrence frequencies, clustering algorithms). These choices, though standard, can introduce a degree of bias or subjectivity in the resulting visualizations and interpretations. Thirdly, restricting the analysis to English-language articles may have led to a language bias, potentially overlooking significant contributions published in other languages, especially considering the rich traditional medicine heritage in non-English speaking countries like China and India. Finally, bibliometric analyses heavily rely on citation counts, which may underestimate the impact of recently published, albeit high-quality, studies due to the time lag inherent in citation accumulation. Despite these limitations, this bibliometric study offers a valuable and comprehensive overview of the research landscape in the field of natural medicines against ionizing radiation.

## Conclusions

This bibliometric study quantitatively and visually analyzed 450 articles on NMs against IR from the WoSCC database, revealing a significant increase in publications, particularly since 2018. India and China emerge as the leading countries, with highly productive institutions and authors, driven by their rich traditional medicine heritage. Enhanced collaboration among countries, institutions, and authors is strongly encouraged to further advance this field.

Research has progressively shifted towards elucidating the molecular mechanisms underlying radioprotection and radiosensitization, involving critical processes such as oxidative stress, inflammation, DNA damage, and apoptosis. The adoption of advanced technologies like network pharmacology, molecular docking, and metabolomics is a prominent trend, enabling a deeper understanding of complex interactions. Future studies should precisely identify the mechanisms of specific NMs and their active ingredients, focusing on key molecular targets, beneficial effects, and potential side effects. Strengthening rigorous clinical studies is essential to translate preclinical findings into validated therapeutic strategies. In conclusion, this bibliometric study defines the overall prospects of the field, offering a valuable reference and inspiration for researchers.

## Data Availability

The raw data supporting the conclusions of this article will be made available by the authors, without undue reservation.

## Glossary

IR: Ionizing Radiation
NMs: Natural Medicines
WoSCC: Web of Science Core Collection
TCM: Traditional Chinese Medicine
ROS: Reactive Oxygen Species
DNA: Deoxyribonucleic acid
WHO: World Health Organization
NRF2: Nuclear factor erythroid 2-related factor 2
NF-κB: Nuclear factor kappa-light-chain-enhancer of activated B cells
HIF-1α: Hypoxia-inducible factor 1-alpha
MAPK: Mitogen-activated protein kinase
JNK: c-Jun N-terminal kinase
TGF-β: Transforming growth factor-beta
